# The Differential Effects of Governmental Direct and Indirect Subsidies on Healthcare Organization’s Profitability in South Korea

**Published:** 2020-04

**Authors:** Sung Man YOON

**Affiliations:** Department of Business Administration, Seoul National University of Science and Technology, Seoul, South Korea

## Dear Editor-in-Chief

In Korea, National Healthcare Insurance is managed by public agencies. These agencies are responsible for determining the insurance unit cost of each medical service and for monitoring the management performance of healthcare organizations (HCOs). These organizations in Korea are classified as not-for-profit (NFP), and the government provides direct and indirect support to ensure their profit to a certain extent (1). In reality, however, many HCOs often abuse or waste government subsidies. The government now is seeking policy solutions to address the agency problem and the challenges in such lax management practices. Some suggest cutting subsidies as a solution (2).

In this context, I investigated the role and effect of direct and indirect government subsidies for HCOs on their management performance. In general, government subsidies are categorized into two types: direct subsidies, such as cash, medical equipment, and facilities, and indirect subsidies, such as tax-exemptions or tax-cuts (3). It is necessary to assess which form of government subsidy is more effective at increasing the profitability of HCOs and strengthening their independence. This is important because the government cannot directly intervene with their decision-making processes regarding internal matters related to management such as investment or employment. Therefore, I assessed the effects of direct or indirect government subsidies on their management (3). In the analysis of the pooled sample shown in Table 1, I found that the indirect government subsidies, such as tax-exemption or tax-credit (INDIRECT; 0.206, *P*<0.001), had a statistically significant positive effect on the dependent variables, net income per healthcare revenue, as financial performance of HCOs. The direct government subsidies (DIRECT) variable did not have any statistically significant effect.

**Table 1:** Ordinary Least Square Regression Results (by Financial Performance)

***Variables***	***Pred. Sign***	***Independent variable: net income per healthcare revenue***		
		Pooled Samples	Profit Orgs.	Loss Orgs.
		Coeff. (t-stat.)	Coeff. (t-stat.)	Coeff. (t-stat.)
Intercept		−0.540(−6.57)^***^	−0.854(−2.41)^***^	−0.688(−7.84)^***^
DIRECT Subsidy	+	0.189(1.28)	2.205(0.98)	0.351(8.07)^***^
INDIRECT Subsidy	+	0.206(4.70)^***^	0.947(2.34)^**^	1.167(6.90)^***^
Log(Asset)	+	0.068(4.77)^***^	0.138(1.91)^*^	0.085(5.62)^***^
LEVERAGE	+	−0.092(−7.63)^***^	0.048(0.40)	0.060(4.93)^***^
AUDITED by CPA [0.1]	+	0.206(5.43)^***^	0.013(0.07)	0.111(2.82)^***^
Government OWN [0,1]	+	0.003(0.10)	0.166(1.13)	0.003(0.11)
Seoul Location [0,1]	−	−0.045(−2.89)^***^	−0.117(−1.97)^*^	−0.066(−3.95)^***^
Year Dummy	+/−	Included	Included	Included
F-Stat.		54.15^***^	2.31^***^	63.90^***^
Adj.R^2^		0.546	0.176	0.623
Observations		311	44	267

Note:

*** < 0.001,

** < 0.05,

* < 0.1 (two-tailed)

This finding suggests that it is more helpful for the government to provide HCOs with indirect subsidies, such as tax exemptions or cuts, than direct subsidies, such as cash or medical equipment, to contribute to their management (4, 5). The analysis of the sample by profit and loss organizations showed that only INDIRECT (0.947, *P*<0.05) had a statistically significant result in the sample of for-profit organizations.

In the analysis of the sample of loss organizations, conversely, both DIRECT (0.351, *P*<0.001) and INDIRECT (1.167, *P*<0.001) demonstrated statistically significant results. These findings indicate that indirect government subsidies, such as tax-exemptions or tax-credits, are primarily more effective for supporting the management performance of for-profit HCOs; however, for loss HCOs showing poor management performance, direct subsidies should also be provided including cash or medical equipment and facilities along with tax benefits to help improve their management performance (6). I conducted another analysis by type of ownership of HCOs according to privately owned organizations (POs) and government-owned organizations (GOs). The results suggested that indirect subsidy (0.083, *P* <0.05), such as tax-exemptions or tax-cuts, is an effective means to support POs management. Conversely, in the case of GOs, direct subsidy (0.202, *P*<0.001), including cash and medical equipment and facilities, was found to be an effective measure to support management performance as well as indirect subsidy (0.188, *P*<0.02).

My findings demonstrate which form of government support—tax-exemption, tax-cut, cash, or medical equipment and facilities—is more effective for improving the management performance of public or NFP HCOs, which play a central role in national public health. I conclude that tax-exemptions or tax-cuts are more effective for for-profit and POs while it is more effective to provide loss and GOs with direct subsidies including cash or medical equipment and facilities along with tax benefits.
